# Advances in Antibody Design and Antigenic Peptide Targeting 2.0

**DOI:** 10.3390/ijms24098033

**Published:** 2023-04-28

**Authors:** Nicole Hartwig Trier, Gunnar Houen

**Affiliations:** Department of Neurology, Rigshospitalet Glostrup, Valdemar Hansensvej 13, 2600 Glostrup, Denmark

Antibodies possess numerous important functions in diagnostics, both as therapeutics and as research tools [1,2]. They can be produced by immunization with proteins, peptide conjugates, etc., or by screening libraries. Specific antibodies can be made using various approaches and can be designed to modify their properties, e.g., by recognizing specific targets, folding into specific structures, increasing immunogenicity and neutralizing effector functions [1,3,4].

This Special Issue of *IJMS*, entitled “Advances in Antibody Design and Antigenic Peptide Targeting”, aims to provide an update as to the status of the current “state-of-the-art” within this field by addressing areas such as antibody design, molecular recognition in antibody-antigen complexes, and the production and use of antibodies, whether recombinant or vaccine-induced as potential therapeutic agents.

Peptides which are suitable for use in generation of peptide antibodies may be identified using bioinformatic tools or phage display [5,6] or carefully selected by studying the protein structure and amino acid sequence, depending on the intended use [7,8]. Following the identification of targets, peptide antibodies are generated using standard procedures, e.g., by immunization of an animal, typically mice, or by phage display [6,7,8,9]. For example, Petry and colleagues successfully examined the reproducibility of phage-displayed peptide selection tools for the production of antibodies, whereas Valdovino-Navaro and coworkers described the selection of a peptide antibody to SARS-CoV-2 SP240, which was isolated from a peptide phage library with high stability [6,10]. In contrast, studies by AlEraky, Trier and Mughal used carefully selected peptides based on amino acid analysis to generate peptide antibodies using common immunization procedures [7,8,9].

As previously mentioned, antibodies play numerous functions. Peptide antibodies generated by immunization have been described as having the function of vaccines for *Helicobacter pylori* infections because there is currently no vaccine available for this microorganism [9]. Moreover, peptide antibodies have been described as neutralizing the interaction of specific viruses [10,11]. For example, peptide antibodies of SP240 have been described as possessing the neutralizing abilities to inhibit binding to live Delta and Omicron SARS-CoV-2 variants, and to the S2 protein of porcine epidemic diarrhea virus in in vivo and in vitro conditions, respectively [10,11]. In addition to this, specific antibodies conjugated with peptides may be used as therapeutics as well. In a study by Ralchev and coworkers, chimeric protein molecules composed of synthetic peptides coupled to a monoclonal antibody were able to inhibit B-cell-specific differentiation to plasmablasts and promote the apoptosis of targeted cells, each of which could aid in the treatment of Hashimoto Thyroiditis [12].

In contrast to their functions as vaccines and therapeutic neutralizing antibodies against virus infections, peptide antibodies may aid in diagnostic assays. For instance, Mughal and collaborators recently described the production of peptide antibodies to the C-terminal of frameshifted calreticulin (CALRmut), which is associated with myeloproliferative diseases [7]. A selected CALRmut peptide antibody with high affinity showed specific reactivity in in vitro and in vivo assays, confirming the applications of the peptide antibody.

Furthermore, peptide antibodies may function as specific research tools. Along these lines, Trier et al., examined antibody cross-reactivities between the P2C coxsackie virus B (CVB) protein and glutamate decarboxylase (GAD)65 by generating peptide antibodies for regions in P2C and GAD65 with 100% sequence similarity (PEVKEK) to examine the potential role of CVB contributing to type 1 diabetes trough molecular mimicry; however, no cross-reactive antibody clones were detected [8]. This is in direct contrast to the results of a recent study by Ehlers and collaborators which generated an antibody to the glucocorticoid receptor; which was found to cross react with the AMP deaminase 2 (AMPD2) and transcription intermediary factor 1-beta (TRIM28) [13]. These findings illustrate one of the potential limitations of using peptide antibodies directed at short amino acid sequences and justify the careful characterization of peptide antibodies prior to use and antibodies in general.

In recent years, antibody-like molecules and nucleic acid-based recognition molecules have been intensely studied. However, there is still a long way to go before these reagents can effectively rival nature’s own reagents: peptides and antibodies.
